# Genetic diversity, morphological traits, quality traits and antioxidants potentiality of *Coccinia grandis* germplasm under rainfed semi-arid region

**DOI:** 10.1038/s41598-023-49091-4

**Published:** 2024-01-09

**Authors:** Lalu Prasad Yadav, K. Gangadhara, V. V. Apparao, Vikas Yadav, D. S. Mishra, A. K. Singh, Jagdish Rane, Prashant Kaushik, P. Janani, Raj Kumar, A. K. Verma, Sanjay Kumar, S. K. Malhotra, Neelam Shekhawat

**Affiliations:** 1 ICAR-Central Horicultulal Experiment Station (CIAH RS), Godhra, 389340 Gujarat India; 2 ICAR-Central Institute for Arid Horticulture, Beechwal, Bikaner, 334006 Rajasthan India; 3 Chaudhary Charan Singh Haryana Agricultural University, Hisar, 125 004 Haryana India; 4 ICAR-Central Potato Research Station, Shillong, 793 009 Meghalaya India; 5 ICAR-Directorate of Knowledge Management in Agriculture, Pusa, 110012 New Delhi India; 6 ICAR-NBPGR Regional Station Jodhpur, C/o CAZRI, Jodhpur, 342003 Rajasthan India

**Keywords:** Evolution, Genetics, Plant sciences

## Abstract

The present study was conducted to evaluate the genetic variability for morphological and qualitative traits of *Coccinia* for development of trait specific lines at ICAR-Central Horticultural Experiment Station (CIAH-RS), Panchmahals (Godhra), Gujarat during 2020–2022. In this study, we evaluated 26 gynoecious accessions to assess the genetic divergence through principal component and cluster analysis. The experiment was carried out in a randomized complete block design with three replications under rainfed semi-arid conditions. High values of PCV and GCV were observed for variables such as NFFP (25.13 and 22.20), PL (23.14 and 20.69), FD (24.01 and 21.46), AFW (22.98 and 20.13), NFPY (26.38 and 24.40), FYP (37.57 and 31.29), FY (35.55 and 33.20), AsC (28.65 and 27.73), Ac (24.32 and 21.06), TSS (37.23 and 35.94), DPPHL (20.71 and 20.38), FRAPL (21.08 and 20.92), TPF(20.81 and 20.45) respectively. High heritability coupled with high genetic advance as per cent of mean was observed for vine length (VL), internodal length (IL), number of female flowers per plant (NFFP), fruit length (FL), peduncle length (PL), fruit diameter (FD), average fruit weight (AFW), number of fruit per plant per year (NFPY), fruit yield per plant (FYP), fruit yield (FY), ascorbic acid (AsC), acidity (Ac), total soluble solids (TSS), total phenols in leaves TPL), total flavonoids in leaves TFL, CUPRAC in leaves (CUPRACL), DPPH in leaves (DPPHL), FRAP in leaves (FRAPL), Total phenols in fruits (TPF), Total flavonoids in fruits (TFF), CUPRAC in fruits (CUPRACF) and DPPH in fruits (DPPHF). The FYP exhibited a significant positive correlation with variables like VL (0.6833), IL (0.2991), NFFP (0.8107), FD (0.5245), AFW (0.6766), NFPY (0.7659), ASC (0.4611), TSS (0.5004) and TPF (0.4281). The estimates of genetic parameters revealed scope for further improvement of fruit yield by selection. Of the eight principal components, PC-I through PC-VIII had eigen values greater than 1 and it accounts 85.02% of the total variation for 26 gynoecious accessions of Ivy gourd. The eigen values of PC-I comprised 5.775% of total variation followed by PC-II (4.250%), PC-III (3.175%), PC-IV (2.588%), PC-V (1.828%), PC-VI (1.447%), PC-VII (1.179%) and PC-VIII (1.013%).The cluster VI and cluster I having highest mean values for most of traits under study. Thus, genotypes from the distinct cluster like cluster VI and I for should be used for selection of parents and varietal improvement for further breeding programme in ivy gourd.

## Introduction

**Figure 1:**
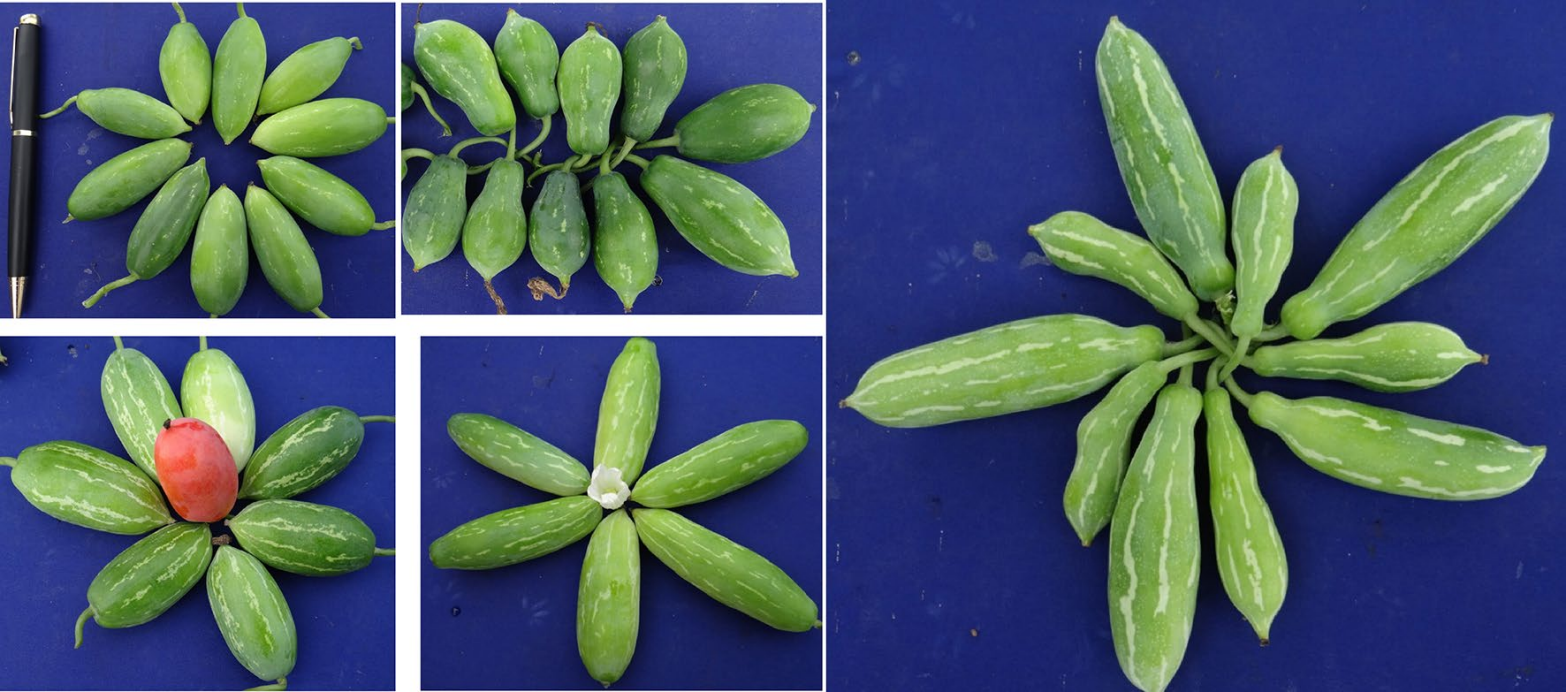
Morphological variability in fruits among promising germplasm at station.

**Figure 2:**
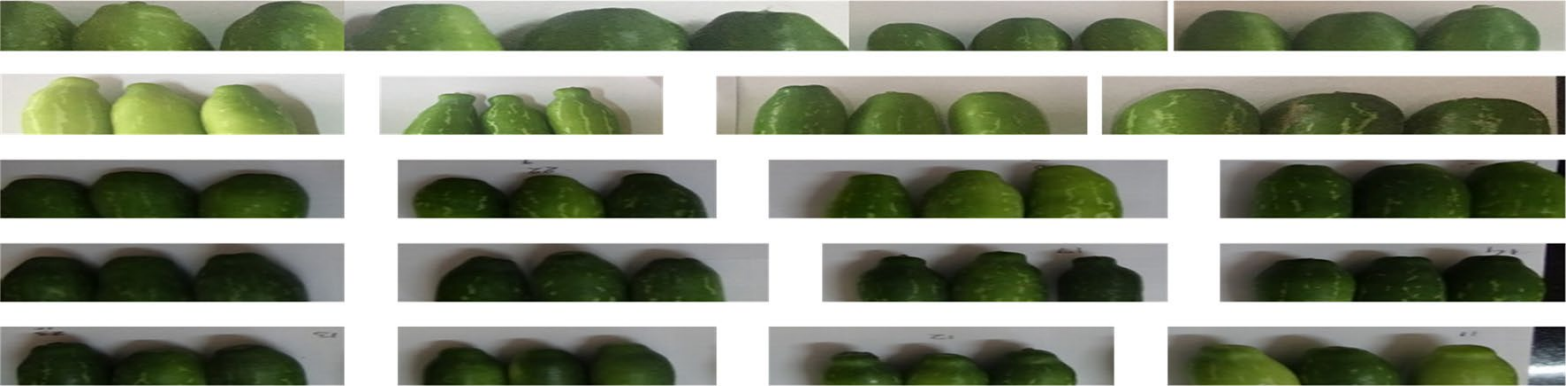
Variation in fruit shoulder appearance in germplasm at station.

**Figure 3:**
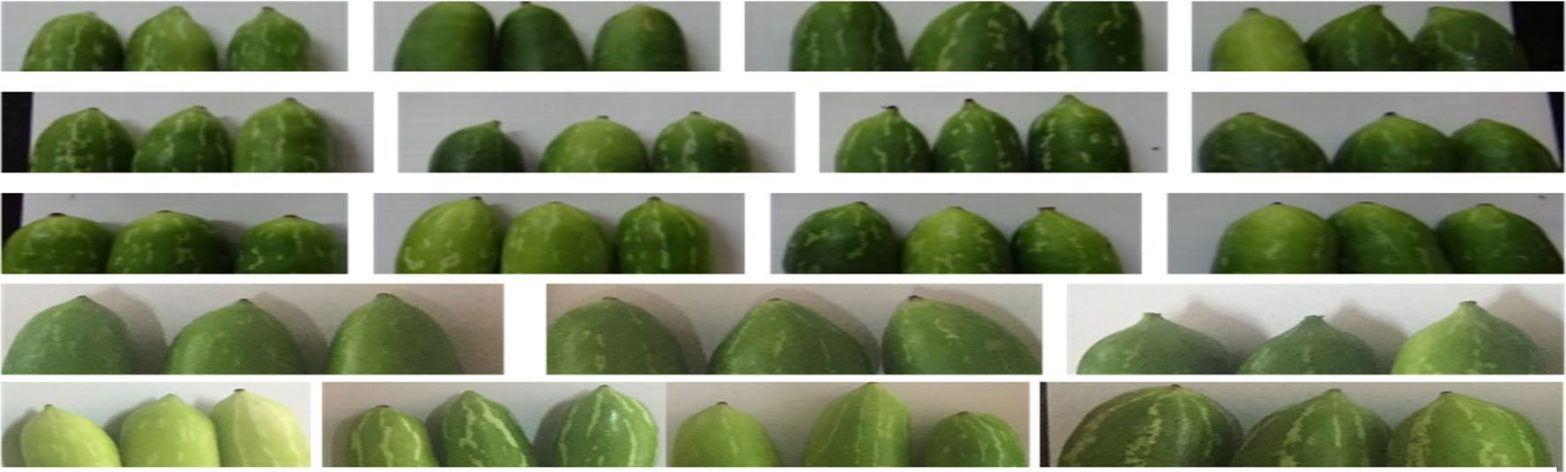
Variation in fruit styler end appearance in germplasm at station.

**Figure 4:**
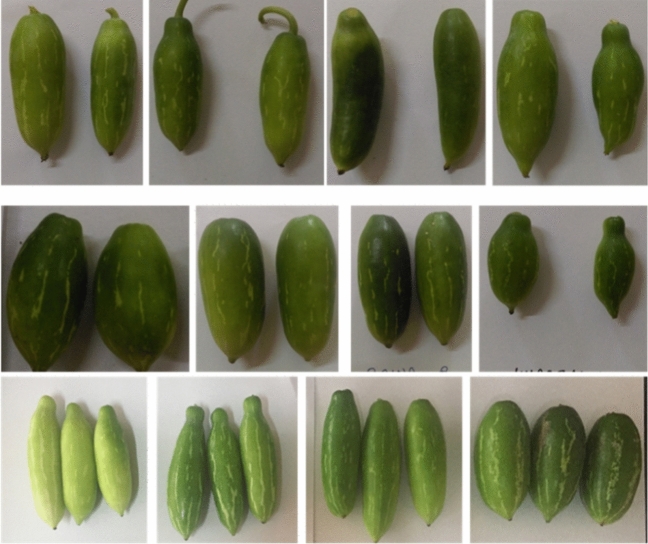
Morphological fruit variability among different germplasm at station.

Ivy gourd, *Coccinia grandis* (L.) Voigt. [Syn. *C. indica* Wight and Arn., *C. cordifolia* (L.) Cogn.] is an underutilized perennial, fast growing, dioecious vegetable of family cucurbitaceae and is known by various names like kundru, tindoli, little gourd and scarlet gourd^1–3^. The *Coccinia* genus comprises 30 species confined to tropical Africa, except *Coccinia grandis*, which occurs wild from Senegal east to Somalia and south to Tanzania, and also in Saudi Arabia, Yemen and India. *Coccinia grandis* is native to India, especially the eastern regions, besides Orissa, Jharkhand, Chhattisgarh, Madhya Pradesh, Gujarat, Maharastra and Andhra Pradesh, where a rich gene pool is available in natural forests as well as in homestead gardens due to its wider adaptability to adverse climatic conditions. The fruit is typically harvested when it is young and tender and can be cooked in a variety of ways, such as stir-frying, boiling, or pickling. Ivy gourd is a good source of vitamins, antioxidants and also contains iron, calcium and zinc^2,4–7^. The experimental site comes under rainfed semi-arid conditions, globally; 22.6 million square kilometers comes under semi-arid region followed by 15.7 million square kilometers of arid region. India is categorized into two zones on semi-arid climatic conditions viz. Thar Desert expands to Rajasthan, Punjab, parts of Uttar Pradesh, Kutch and Saurashtra. The another is located in the south and covers the Deccan plateau, the Coimbatore plateau and the utmost southeast region of Madras (Ramanathapuram and Tirunelveli area). The two zones are delineated by a narrow, humid region encompassing the Satpura range and the Tapti River plain (3). Its tender fruits and shoots are used for cooking and are rich sources of carbohydrates, protein, antioxidants and vitamins. It is widely used in the traditional treatment of diabetes, bronchitis, skin disorders, small pox, ring worm, scabies, ulcers, gonorrhoea, constipation, insect bites, allergy, eye infections, gonorrhoea, syphilis, liver weakness and fever and prescribed in traditional medicine for different ailments; widely used in Ayurvedic, Unani and Siddha practice in the indian subcontinent. It also has hypolipidemic, antimutagenic, hypoglycemic and anti-inflammatory activities^3,6–10^. Understanding the nutritional importance and other advantages, it is a climber that propagates vegetatively has a wide range of phylogenetic, morphological and ecological diversity. The diversity in vegetatively propagated crops might be due to the diversity of their ancestors, the diverse ecologies of the crop populations themselves and the intricate mix of selection pressures acting on the parts harvested and on the parts used by humans to make clonally propagate, resulting in complex and diverse evolutionary trajectories under domestication. The domestication of cucurbitaceous vegetable crops has involved different morphological traits including fruit shape, less bitter flesh, larger and fewer seeds, and large fruit size, resulting in high genetic diversity within and among cultivated species^11^. The crop plays an important role in the local diet of rural and per urban areas mainly in tribal arid, semi-arid and humid regions of India. Variability for morphological and qualitative traits including antioxidants among *C. grandis* germplasm are frequently used in breeding programs for developing cultivars with consumer liking is a prerequisite to efficiently manage and utilize germplasm. The PCA and clustering analysis of morphological characterization are relatively inexpensive and easy to carry out for conservation of genetic resources, identification of characters amenable to genetic improvement and selection of high yielding genotypes^12^. The success of good breeding and selection of promising germplasm depends on the genetic variability present in the germplasm population and the variation in the population helps to identify suitable germplasm for vital traits and conserve and classify genetic variation in the plant germplasm. As a result, the degree of genetic diversity in the base population influences the generation of high-yielding genotypes in crop improvement^5,13^. With the considerations mentioned above, the current study was conducted to evaluate the genetic variability for morphological and qualitative traits of *Coccinia* germplasm for development of trait specific lines. Further, this investigation supported in the development of two varieties namely CHESIG-2 as Thar Sadabahar and CHESIG-7 as Thar Dipti at ICAR-CIAH, Bikaner under rainfed semi-arid conditions (Figs. 1, 2, 3 and 4).

## Materials and methods

### Plant material

A set of 34 gynoecious accessions, which included three cultivars of *C. grandis,* were planted through stem cutting and maintained. Among these, 26 gynoecious accessions of cultivated species *C. grandis* were evaluated in a randomized complete block design with three replications under rainfed semi-arid conditions during 2020–2022 at Vegetable Experimental Farm, ICAR-Central Horticultural Experiment Station (CIAH RS), Panchmahals (Godhra), Gujarat (Table 1 and Figs. 1, 2, 3 and 4). The average maximum and minimum temperature ranged between 28.4–46.5 °C and 12.7–26.7 °C, respectively, and total annual minimum and maximum rainfall ranged from 293.24 to 941.25 mm with relative humidity 27.55–92.50 per cent during the period under study, which is favorable for ivy gourd cultivation. However, the site’s annual water requirement or potential evapo-trasnspiration is approximately 1500 mm^14^. Standard cultural practices and production technology including planting, pruning, cultural practices, fertilizer application and protection measures were followed^15–17^.

**Table 1:** List of *Coccinia* germplasm, collection site from Gujarat and their morphological traits.

Sr No.	Germplasm	Collection Site	Leaf shape	Fruit shape	Stripness
1.	CHESIG-1	Vav, Jhambhughoda	Pentalobed	Round oblong	Continuous
2.	CHESIG-2	Vav, Jhambhughoda	Pentalobed	Round oblong	Discontinuous
3.	CHESIG-3	Baina, Devgarh Baria	Pentalobed	Round	Continuous
4.	CHESIG-4	Rampur, Morva Hadaf	Pentalobed	Oblong	Discontinuous
5.	CHESIG-5	Rampur, Morva Hadaf	Pentalobed	Oblong	Discontinuous
6.	CHESIG-6	Rampur, Morva Hadaf	Trilobed	Round oblong	Discontinuous
7.	CHESIG-7	Rampur, Morva Hadaf	Trilobed	Round oblong	Stripless
8.	CHESIG-8	Dantol, Ghogumba	Pentalobed	Shouldered oblong	Continuous
9.	CHESIG-9	Baina, Kalol	Pentalobed	Spindle shape	Discontinuous
10.	CHESIG-10	Rampur, Morva Hadaf	Cordate	Pear shape	Sparse stripes
11.	CHESIG-11	Kharsaliya, Kalol	Pentalobed	Round oblong	Discontinuous
12.	CHESIG-12	Kharsaliya, Kalol	Pentalobed	Oblong	Discontinuous
13.	CHESIG-13	Kharsaliya, Kalol	Pentalobed	Oblong	Discontinuous
14.	CHESIG-14	Kharsaliya, Kalol	Pentalobed	Round oblong	Discontinuous
15.	CHESIG-15	Kharsaliya, Kalol	Pentalobed	Shouldered oblong	Continuous
16.	CHESIG-16	Baina, Devgarh Baria	Pentalobed	Oblong	Continuous
17.	CHESIG-17	Baina, Devgarh Baria	Pentalobed	Round oblong	Discontinuous
18.	CHESIG-18	Baina, Devgarh Baria	Pentalobed	Oblong	Discontinuous
19.	CHESIG-19	Baina, Devgarh Baria	Trilobed	Round oblong	Stripless
20.	CHESIG-20	Timarva, Dahod	Pentalobed	Round oblong	Continuous
21.	CHESIG-21	Timarva, Dahod	Pentalobed	Round oblong	Continuous
22.	CHESIG-22	Vav, Jhambhughoda	Pentalobed	Spindle shape	Discontinuous
23.	CHESIG-23	Vav, Jhambhughoda	Pentalobed	Oblong	Discontinuous
24.	CHESIG-24	Vav, Jhambhughoda	Pentalobed	Shouldered oblong	Continuous
25.	CHESIG-25	Kotda, Bhavnagar	Pentalobed	Round oblong	Continuous
26.	CHESIG-26	Kotda, Bhavnagar	Pentalobed	Oblong	Discontinuous

### Data collection and sample preparation

**Figure 5:**
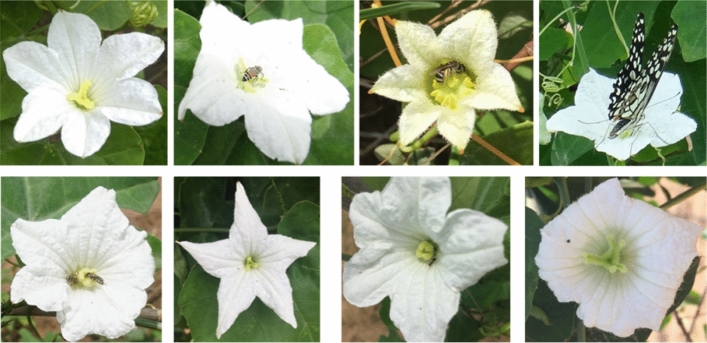
Variability in ivy gourd flowers and their pollinators at station.

The evaluation for observation and determination of morphological parameters, quality traits and antioxidant potentiality was performed in three replications at the station (Tables 2, 3, 4). The flower variability (Fig. 5) was also observed. The border plants at both ends of the plots were discarded. The morphological data were recorded on eight plants selected randomly from each replication. The sample of marketable fruits and tender leaves were harvested from pest and disease free healthy plants for different observations and analysis. The samples were washed with tap water and excess water was drained. The fresh samples of fruit were used for the determination of TSS, ascorbic acid and acidity while, fresh samples of fruits and tender leaves were used for the determination of total phenols, total flavonoids, CUPRAC, DPPH and FRAP.

**Table 2:** Cluster mean analysis for twenty five traits in 26 *coccinia* accession.

S.N.	Character	Clusters					
		I	II	III	IV	V	VI
1.	VL	349.6	307.767	294.15	296.58	335.275	449
2.	IL	8.3	7.367	7.75	6.61	6.45	7.45
3.	LL	7.767	7.767	8.4	7.84	7.075	8.3
4.	LW	7.167	7.367	7.725	7.18	6.525	7.8
5.	NFFP	1148.333	1621.333	1372	941.6	1453.75	1318.5
6.	FL	6.1	4.533	4.95	5.1	4.7	6.35
7.	PL	6.033	5.033	7.325	5.91	5.625	5.85
8.	FD	3.067	2.667	2.3	2.22	2.25	2.8
9.	AFW	18.333	15.667	14.225	14.56	15.25	24.75
10.	NFPY	1097.533	1465.667	1318.2	844.7	1390.75	1290
11.	FYP	20.28	22.899	18.878	12.406	21.343	31.418
12.	FY	16.483	18.265	15.351	10.084	17.298	26.09
13.	AsC	25.94	47.287	29.385	31.05	31.1	47.325
14.	Ac	0.104	0.128	0.083	0.125	0.117	0.137
15.	TSS	1.767	1.733	2	1.56	2.125	2.6
16.	TPL	22.453	18.147	13.308	17.676	19.633	17.885
17.	TFL	11.313	9.25	7.779	8.045	7.26	6.54
18.	CUPRACL	32.84	36.899	23.462	27.427	28.576	29.38
19.	DPPHL	17.67	18.707	14.293	19.422	20.883	18.108
20.	FRAPL	30.282	41.536	27.314	30.247	30.458	29.637
21.	TPF	14.338	13.347	10.936	10.593	13.81	12.095
22.	TFF	7.08	6.263	5.085	5.447	4.528	6.615
23.	CUPRACF	24.32	20.33	17.293	18.147	19.123	17.64
24.	DPPHF	21.246	23.683	19.873	18.667	19.628	18.047
25.	FRAPF	29.197	29.644	25.011	25.518	26.035	25.817

**Table 3:** Cluster composition of twenety six Ivy gourd genotypes in to six clusters.

Sr.N.	Clusters	No. of Genotypes	Name of genotypes
1.	I	03	CHESIG-1, CHESIG-3 and CHESIG-9
2.	II	03	CHESIG-10, CHESIG-6 and CHESIG-7
3.	III	04	CHESIG-11, CHESIG-18, CHESIG-4 and CHESIG-5
4	IV	10	CHESIG-12, CHESIG-13, CHESIG-16, CHESIG-17, CHESIG-20, CHESIG-21, CHESIG-22, CHESIG-24, CHESIG-25 and CHESIG-26
5.	V	04	CHESIG-14, CHESIG-15, CHESIG-19 and CHESIG-23
6.	VI	02	CHESIG-2 and CHESIG-8

**Table 4:** Salient features of promising germplasm of *Coccinia* identified during the study.

Sr No.	Germplasm	IC number	Salient features
1.	CHESIG-2 (Thar Sadabahar)	IC632331	Dark green colour with discontinuous strips, round oblong fruit shape without neck, round the year fruit production
2.	CHESIG-3	IC632332	Round shape fruit, continuous white stripes
3.	CHESIG-4	IC632333	Deep pentalobbed leaf, medium oblong, green colour fruit with discontinuous stripes
4.	CHESIG-7 (Thar Dipti)	IC632334	Dark green stripeless fruit appearance, trilobe leaf shape, small-medium size fruit and pointed styler end
5.	CHESIG-8	IC632335	Shouldered oblong shape fruit, light green fruit colour having continuous white stripes and low TSS
6.	CHESIG-9	IC632336	Less seeded, spindle shape fruit having natural green colour, pointed styler end and deep shoulder length
7.	CHESIG-10	IC632337	Cordate leaf shape, small size green colour pear shape fruit with sparse white stripes, suits for winter season

### Determination of total soluble solids (TSS) and acidity

TSS and acidity were analyzed as suggested and described^18^.

### Determination of ascorbic acid content

Ascorbic acid content was determined in accordance with the dinitrophenylhydrazine (DNPH) method. Fresh sample was homogenized in mortar pestle, with 20 mL of a mixture of 6% (w/v) metaphosphoric acid in 2 mol/L acetic acid. The mixture was centrifuged at 10,000 rpm for 15 min at 4 °C. The supernatant was filtered through Whatman filter paper (No. 1). The extract was titrated against dye and note the titrate value when pink colour was appeared. The value was expressed as mg/100 g fw^14^.

### Determination of total phenolics and total flavonoids

Total phenolics were estimated using Folin–Ciocalteu reagent^19^. To 100 µL of the sample extract (80% ethanol) 2.9 mL of deionized water, 0.5 mL of Folin–Ciocalteu reagent and 2.0 mL of 20% Na_2_CO_3_ solution were added. The mixture was allowed to stand for 90 min and absorption was measured at 760 nm against a reagent blank in UV–Vis spectrophotometer. Results were expressed as Gallic acid equivalent (mg GAE/100 g fw). Total flavonoids were analyzed using aluminum chloride method^20^. An aliquot of 1 mL of extract was added to 10 mL of volumetric flask containing 4 mL of distilled water, 0.3 mL portion of 5% NaNO_2_ and 0.3 mL portion of 10% AlCl_3_·6H2O. The mixture was allowed to stand for 6 min at room temperature. 2 mL of 1 N NaOH was added and the solution was diluted to 10 mL with distilled water. The absorbance of the solution versus a blank at 510 nm was measured immediately. The results were expressed as Catechin equivalent (mg CE/100 g fw).

### Determination of antioxidant activity

The cupric ion reducing antioxidant capacity and ferric reducing antioxidant potential of the fruits and leaveswere determined according to the method proposed by^21,22^, respectively. The FRAP reagent included 300 mM acetate buffer, pH 3.6, 10 mM TPTZ in 40 mM HCl and 20 mM FeCl3 in the ratio 10:1:1 (v/v/v). Three ml of the FRAP reagent was mixed with 100 µL of the sample extract in a test tube and vortexed in the incubator at 37 °C for 30 min in a water bath. Reduction of ferric-tripyridyltriazine to the ferrous complex formed an intense blue colour which was measured; at a UV–vis spectrophotometer (Varian Cary 50) at 593 nm. The CUPRAC according to the protocol 0.1 mL of sample extract was mixed with 1 mL each of CuCl_2_ solution (1.0 × 10^2^ mol/L), neocuproine alcoholic solution (7.5 × 10^3^ mol/L), and NH4Ac (1 mol/L, pH 7.0) buffer solution and 1 mL of water to make the final volume 4.1 mL, After 30 min, the absorbance was recorded at 450 nm against the reagent blank. Standard curve was prepared using different concentration of Trolox. Free radical scavenging assay was assessed by the measurement of the scavenging ability of plant extract toward the stable radical DPPH^23^. A 3.9 mL aliquot of a 0.0634 mM of DPPH solution, in methanol (95%) was added to 0.1 mL of each extract and shaken vigorously. Change in the absorbance of the sample extract was measured at 515 nm for 30 min till the absorbance reached a steady state. The percentage inhibition of DPPH of the test sample and known solutions of Trolox were calculated by the following formula

$$\% Inhibition = 100 \times \left( {A_{0} - A} \right)/A_{0}$$

where A_0_ was the beginning absorbance at 515 nm, obtained by measuring the same volume of solvent, and A was the final absorbance of the sample extract at 515 nm. Methanol (95%) was used as a blank. Results were expressed as µmol Trolox equivalent (TE)/g fw.

### Statistical analysis

The data and plot analysis were carried out using various packages of *Rstudio* version 2023.3.0 + 386. The latest available versions of *FactoMineR*, *factoextra*, and *ggplot2* were used for Principal component analysis^24,25^**.** Cluster analysis was carried out with the help of the cluster, *factoextra*, *dendextend*, and *ggplot2* packages^26^. The variability analyzed by variability and *agricolae* and correlation by *corrplot* (46).

The collected accessions of gynoecious *coccinia* planted through stem cutting and maintained in field repository at ICAR-Central Horticultural Experiment Station (CIAH RS), Panchmahals (Godhra), Gujarat after following all methods in accordance with relevant guidelines of ICAR-NBPGR, New Delhi, India (http://www.nbpgr.ernet.in).

## Results and discussion

Analysis of Variance (ANOVA) for 25 different traits in 26 *coccinia* gynoecious accessions were presented in Table 5 (***0.1% level of significance).

**Table 5:** Analysis of variance for 25 quantitative and qualitative traits in 26 *coccinia* gynoecious accessions (Mean Squares).

	df	VL	IL	LL	LW	NFFP	FL	PL	FD	AFW	NFPY	FYP	FY
Replication	2	794.923	0.012	0.001	0.029	5851.192	0.051	0.014	0.064	0.213	0.379	0.379	0.278
Genotypes	25	11,380.37***	3.34***	1.2***	0.89***	239,793.19***	2.78***	4.99***	0.88***	34.11***	113.57***	113.57***	77.17***
Error	50	1304.35	0.049	0.057	0.191	20,565.472	0.279	0.385	0.069	3.132	14.57	14.575	3.607
S.Em +		20.852	0.128	0.138	0.252	82.796	0.305	0.358	0.151	1.022	65.811	2.204	1.097
C.D. at 5%		59.229	0.364	0.392	0.716	235.184	0.866	1.017	0.430	2.902	186.937	6.261	3.115
C.D. at 1%		78.964	0.486	0.522	0.955	313.546	1.154	1.356	0.573	3.869	249.223	8.347	4.153

	AsC	Ac	TSS	TPL	TFL	CUPRACL	DPPHL	FRAPL	TPF	TFF	CUPRACF	DPPHF	FRAPF
Replication	1.608	0.00002	0.017	0.036	0.108	0.980	0.945	0.054	0.096	0.016	0.033	0.937	0.362
Genotypes	262.22 ***	0.002***	1.34***	30.42***	7.44***	84.48***	42.97***	127.53***	18.29***	3.16***	17.71***	16.53***	14.72***
Error	5.758	0.00017	0.032	0.988	0.102	0.674	0.460	0.642	0.219	0.072	1.220	0.609	0.300
S.Em +	1.385	0.008	0.103	0.574	0.184	0.474	0.392	0.462	0.270	0.154	0.638	0.451	0.316
C.D. at 5%	3.935	0.021	0.292	1.630	0.523	1.346	1.112	1.314	0.767	0.439	1.812	1.280	0.899
C.D. at 1%	5.246	0.029	0.390	2.173	0.697	1.795	1.483	1.751	1.023	0.585	2.415	1.706	1.198

VL—vine length, IL—internodal length, LL—leaf length, LW—leaf width, NFFP—number of female flowers per plant, FL—fruit length, PL—Peduncle length, FD—fruit diameter, AFW—average fruit weight, NFPY—number of fruit per plant per year, FYP—fruit yield per plant, FY—fruit yield, AsC—ascorbic acid, Ac—acidity, TSS—total soluble solids, TPL—total phenols in leaves, TFL—total flavonoids in leaves, CUPRACL—CUPRAC in leaves, DPPHL—DPPH in leaves, FRAPL—FRAP in leaves, TPF—total phenols in fruits, TFF—total flavonoids in fruits, CUPRACF—CUPRAC in fruits, DPPHF—DPPH in fruits, FRAPF—FRAP in fruits.

***0.1% level of significance.

### Variations among germplasms for different morphological, fruit morphometric and yield traits

The analysis of variance revealed significant variation among the 26 coccinia gynoecious accessions including released Indian varieties and breeding lines for 25 characters (Table 5). This indicated the presence of high degree of variation within the genotypes. One approach to assess variability is by examining the range of variations. Range of variation observed for all the traits in the present study (Table 6) indicated the presence of sufficient amount of variation among the genotypes for all the characters. Diversity in plant genetic resources provides the opportunity to breeders for development new and improved varieties with desirable traits, which include both farmer-preferred traits and breeder preference traits^27^. The results indicated that all traits differed significantly among the 26 coccinia gynoecious accessions. The longest vine length was observed in CHESIG2 (487.40 cm), while the shortest was CHESIG-13(180 cm). Internodal length ranged from 5.80 cm in CHESIG-13 to 9.50 cm in CHESIG-4. The maximum mean leaf length and width exhibited in CHESIG-11 (9.10 and 8.20 cm). These findings are in line with^11,28–30^ who also found significant differences in morphological traits of ivy gourd germplasm. The number of female flowers per plant is an important yield-contributing trait, range from 738 (CHESIG-20) to 1684 (CHESIG-6). According^31^, low female flower numbers reduces the productivity of ivy gourd cultivation.

**Table 6:** Range, mean, estimates of components of variance, heritability and genetic advance for growth, yield and quality parameters in Ivy gourd.

Character	Range		Mean	GCV	PCV	Heritability	Genetic advance at 5%	Genetic advance as % mean
	Min	Max						
VL	180.00	487.40	321.29	18.04	21.25	72	101.32	31.54
IL	5.80	9.50	7.11	14.75	15.08	96	2.11	29.73
LL	6.60	9.10	7.83	7.89	8.46	87	1.19	15.16
LW	6.00	8.20	7.23	6.71	9.03	55	0.74	10.27
NFFP	738.00	1684.00	1217.88	22.20	25.13	78	491.93	40.39
FL	3.80	7.50	5.16	17.71	20.46	75	1.63	31.60
PL	3.60	8.20	5.99	20.69	23.14	80	2.28	38.12
FD	1.50	4.40	2.43	21.46	24.01	80	0.96	39.51
AFW	11.60	28.20	15.96	20.13	22.98	77	5.80	36.33
NFPY	658.00	1570.00	1136.63	24.40	26.38	86	528.47	46.49
FYP	9.28	31.62	18.36	31.29	37.57	69	9.86	53.69
FY	7.51	26.86	14.92	33.20	35.55	87	9.52	63.85
AsC	21.02	51.35	33.34	27.73	28.65	94	18.44	55.30
Ac	0.06	0.16	0.12	21.06	24.32	75	0.04	37.57
TSS	1.00	3.20	1.84	35.94	37.23	93	1.31	71.49
TPL	11.87	24.19	17.93	17.47	18.33	91	6.15	34.31
TFL	5.97	12.06	8.28	18.89	19.28	96	3.16	38.13
CUPRACL	20.05	40.06	28.86	18.31	18.53	98	10.76	37.28
DPPHL	11.65	26.84	18.47	20.38	20.71	97	7.63	41.32
FRAPL	20.54	46.16	31.09	20.92	21.08	99	13.30	42.77
TPF	8.04	16.27	12.01	20.45	20.81	97	4.97	41.37
TFF	3.76	7.26	5.62	18.06	18.68	94	2.02	35.99
CUPRACF	15.21	25.31	19.09	12.28	13.58	82	4.37	22.89
DPPHF	16.02	26.24	19.83	11.62	12.27	90	4.50	22.67
FRAPF	22.06	31.27	26.44	8.29	8.55	94	4.38	16.58

GCV— genotypic coefficient of variation, PCV—phenotypic coefficient of variation, H^2^b— broad sense heritability, GA— genetic advance, GAM—genetic advance as percentage of mean.

A wide range of variation was observed for fruit characters. The fruit length ranged from (3.80 cm in CHESIG-16 to 7.50 cm in CHESIG-2) and fruit diameter (1.50–4.40 cm in CHESIG-13 and CHESIG-3), peduncle length (3.6 cm in CHESIG-10 to 8.2 cm in CHESIG-11). Fruit weight ranged from 11.60 g in CHESIG-16 to 28.20 g in CHESIG-2, with an average of 15.96 g. In *coccinia* breeding programmes, the number of fruits per plant is an important yield-contributing trait.The number of fruit per plant per year ranged from 658 in CHESIG 20 to 1570 in CHESIG 6, with a mean value of 1136.63 respectively. *Coccinia* CHESIG-2 genotypes had distinctly higher fruit yield 31.62 kg/plant and 26.86 t/ha more fruit than the other genotypes. In contrast, CHESIG-17 produced the lowest fruit yield of 9.28 kg/plant and 7.51 t/ha. These genotypes can be used in *cocinia* breeding programmes to develop cultivars with desired traits. Significant variation of fruit characters was also reported in ivy gourd^4,11,28–30^ and in cucumber ^27,32^.

Estimates of components of variance, heritability and genetic advance for growth, yield and quality parameters in ivy gourd were given in Table 6. Estimate of phenotypic coefficients of variation (PCV) ranged between 8.46% for leaf length to 37.57% for fruit yield per plant and genotypic coefficient of variation (GCV) ranged between 6.71% for LW and 35.94% for TSS (Table 6). High PCV and GCV were recorded for NFFP (25.13 and 22.20), PL (23.14 and 20.69), FD (24.01 and 21.46), AFW (22.98 and 20.13), NFPY (26.38 and 24.40), FYP (37.57 and 31.29), FY (35.55 and 33.20), AsC (28.65 and 27.73), Ac (24.32 and 21.06), TSS (37.23 and 35.94), DPPHL (20.71 and 20.38), FRAPL (21.08 and 20.92), TPF (20.81 and 20.45) respectively. whereas, high PCV and moderate GCV was observed in VL(21.25 and 18.04) and FL(20.46 and 17.71). The presence of high variability within the genotypes offers better scope for improvement through selection. These results indicating maximum amount of variability present in the germplasm for these characters. PCV is slightly higher than GCV for most of traits under study, indicating less environmental influence. Similar results were given in ivy gourd^4,11,29,30^. Moderate PCV and GCV were recorded for the characters like IL (15.08 and 14.75), TPL (18.33 and 17.47), TFL (19.68 and 18.89), CUPRACL (18.53 and 18.31), CUPRACF (21.08 and 20.92), TFF (18.28 and 18.06), DPPHF (12.27 and 11.62). These results explain the existence of limited variability or low genetic variability in the germplasm evaluated for the trait. This necessitates need for generation of new variability for these characters. Low PCV and GCV was recorded for LL (8.46 and 7.89), LW (9.03 and 6.71), and FRAPF (8.55 and 8.29) which Indicates the existence of limited variability or low genetic variability in the germplasm evaluated for these traits and selection based on these traits would be ineffective^33,34^. Genetic variability in terms of PCV and GCV were high for yield per plant (27.56 and 23.87%, respectively). High heritability in broad sense combined with high genetic advance was recorded for number of fruits per plant (94.39 and 38.57%) followed by fruit weight (93.36 and 32.61%) are the indicative of preponderance of additive and additive × additive type of gene interaction in *C. Grandis* ^33^. Similarly in cucumber, the highest estimates (> 20%) of PCV and GCV were observed for the internode distance, average fruit weight, number of fruits per plant, and yield per plant, indicating a wide range of variations^27^.

The dominance of selection for any trait depends not only on the amount of phenotypic and genotypic variability but also on estimates of broad sense heritability. High heritability in capacious sense is effective in elucidating desirable trait for selection and enables the breeder to select superior genotypes on the basis of phenotypic expression of quantitative traits^4,13,30,35^. In the present investigation, high heritability was recorded for all traits. FRAPL exhibited highest broad heritability (99%) followed by CUPRACL (98%), DPPHL and TPF (97%), IL as well as TFL (96%). It alluding that these traits are less influenced by environmental factors and are under the control of additive gene effect and identification for advancement of such traits would be rewarding. Heritability estimates in confluence with genetic enhancements are more dominant and reliable in predicting the advancement through selection^36^. So far as the units of measurements influence the magnitude of genetic advance (GA), the GA as per cent of mean is contemplated as an imperious selection parameter. Genetic advance as per cent mean (GAM) is highest for most of the traits except LL, LW and FRAPF. High heritability results in cucumber for different traits like vine length (94.41%), internodes distance (92.07%), the number of primary branches plant^−1^ (84.17%), fruit weight (93.99%), fruit length (87.61%), number of fruits plant^−1^ (84.88%), and yield plant^−1^ (89.9%) were observed^27^. High heritability associated with high Genetic advance as per cent mean was observed for VL, IL, NFFP, FL, PL, FD, AFW, NFPY,FYP, FY, AsC, Ac, TSS, TPL, TFL, CUPRACL, DPPHL FRAPL, TPF, TFF, CUPRACF, DPPHF, CUPRACF and DPPHF indicated additive gene action, making selection for this trait will be more efficacious^36,37^. High heritability along with low GAM was recorded for LL, LW and FRAPF due to non-additive gene action and direct selection for these parameters will be less efficacious. The similar results were recorded by^4^ in ivy gourd and spine gourd^38^.

**Figure 6:**
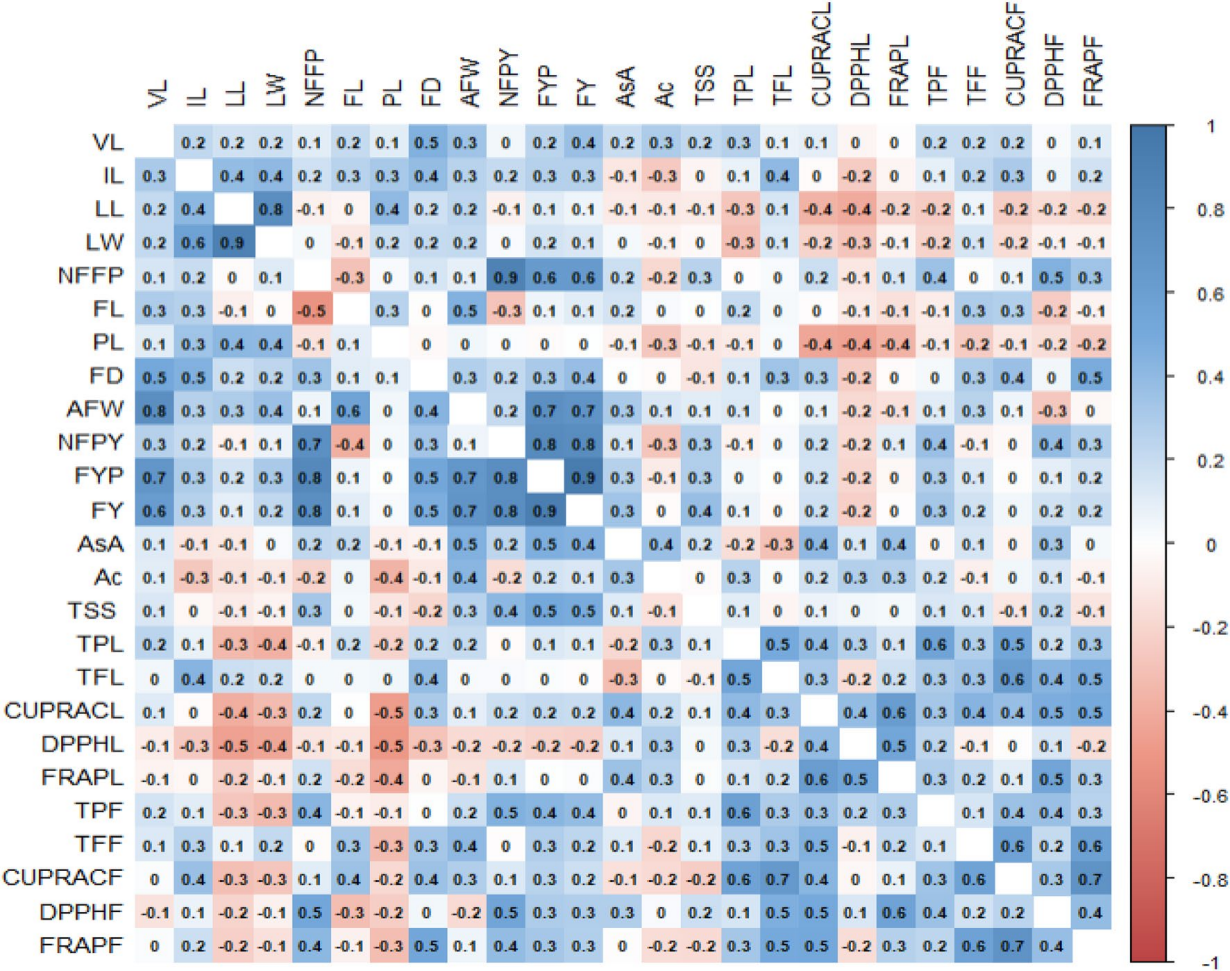
Graphical representation of correlation coefficients for growth, yield and quality traits in ivy gourd (Diagonally above is phenotypic and below is genotypic correlation coefficients).

The genotypic and phenotypic correlation coefficients were worked out for 25 morphological, yield and fruit quality characters of the 26 *coccinia* germplasm (Table 7 and Fig. 6). It was evident from the table that the values of genotypic correlation coefficient were greater than the values of phenotypic correlation co efficient for most of the characters, which indicate a strong inherent association between various traits. In phenotypic correlation (Table 7), fruit yield per plant showed significant positive correlation with VL (0.2426), IL (0.2639), NFFP (0.6338), FD (0.3077), AFW (0.7452), NFPY (0.795), ASC (0.2636), TSS (0.2819), TPF(0.2892). Whereas, fruit yield per plant showed phenotypic negative correlation with PL (− 0.0192), Ac (− 0.0975), DPPHL (− 0.1968), FRAPL (− 0.0975) and (0.0053). Similar findings were reported by^4,5,8,11,13,30,38^. In genotypic level (Table 6), fruit yield per plant showed significant positive correlation with VL (0.6833), IL (0.2991), NFFP (0.8107), FD (0.5245), AFW (0.6766), NFPY (0.7659), ASC (0.4611), TSS (0.5004), TPF(0.4281).Whereas, fruit yield per plant showed genotypic negative correlation with DPPHL (-0.2084). In line with our findings, the phenotypic correlation studies in cucumber showed that fruit yield plant^−1^ exhibited a positive and significant correlation with fruits plant^−1^, fruit length, fruit weight, fruit width, branches plant^−1^ and plant height ^10^. Many researchers reported in earlier findings that leaves are important trait for plants photosynthetic performance, architecture and yield potential, thus, a good trait to be targeted for breeding programme for development and selection of targeted trait variety^12^. The characters like showed high positive correlation both in phenotypic as well as genotypic level, which indicating that there was simultaneous selection for these characters might bring an improvement in fruit yield of plant. Similar results were reported by^4,5,38–41^.

**Table 7:** Phenotypic and genotypic correlation coefficients for growth, yield and quality traits in ivy gourd.

Traits	VL	IL	LL	LW	NFFP	FL	PL	FD	AFW	NFPY	FYP	FY	AsA
VL	1	0.2267*	0.1694	0.1788	0.1056	0.202	0.1052	0.4569**	0.3425**	0.0462	0.2426*	0.3631**	0.1916
IL	0.3073	1	0.3867**	0.403**	0.1761	0.2532*	0.3176**	0.3814**	0.2506*	0.2015	0.2639*	0.2637*	− 0.116
LL	0.2165	0.4176*	1	0.7605**	− 0.0672	− 0.0415	0.4012**	0.1748	0.2323*	− 0.0629	0.1041	0.0521	− 0.0946
LW	0.2153	0.5771**	0.899**	1	0.0311	− 0.115	0.1834	0.2088	0.211	0.0221	0.1587	0.1328	0.033
NFFP	0.1476	0.1906	− 0.019	0.1128	1	− 0.2928**	0.021	0.1464	0.084	0.8747**	0.6338**	0.6314**	0.1849
FL	0.2965	0.2685	− 0.0788	− 0.0147	− 0.5215**	1	0.2516*	− 9.00E− 04	0.4586**	− 0.2842*	0.0563	0.1085	0.1552
PL	0.1004	0.3405	0.4197*	0.4052*	− 0.1343	0.0933	1	− 0.0278	0.0403	0.0117	− 0.0192	0.0019	− 0.0576
FD	0.5146**	0.4579*	0.1854	0.2304	0.3487	0.1089	0.0885	1	0.2705 *	0.2168	0.3077 **	0.3718 **	0.0036
AFW	0.7516 **	0.264	0.2973	0.3698	0.0524	0.5618 **	0.0463	0.4332 *	1	0.2042	0.7452 **	0.7017 **	0.3017 **
NFPY	0.2632	0.209	− 0.0567	0.0685	0.7404 **	− 0.3818	0.0308	0.3337	0.053	1	0.795 **	0.7714 **	0.1279
FYP	0.6833 **	0.2991	0.1576	0.3068	0.8107 **	0.0534	0.0025	0.5245 **	0.6766 **	0.7659 **	1	0.9411 **	0.2636 *
FY	0.6203 **	0.269	0.0902	0.227	0.7534 **	0.1019	0.0266	0.4736 *	0.6924 **	0.7699 **	0.9161 **	1	0.3405 **
AsA	0.1151	− 0.1169	− 0.102	0.0223	0.2206	0.2026	− 0.0741	− 0.0531	0.4523 *	0.2326	0.4611 *	0.4429 *	1
Ac	0.1071	− 0.2606	− 0.1324	− 0.1005	− 0.2037	0.0433	− 0.363	− 0.1041	0.4221 *	− 0.17	0.1548	0.1305	0.323
TSS	0.1354	− 0.035	− 0.0991	− 0.0562	0.3231	0.0321	− 0.121	− 0.1956	0.2572	0.4107 *	0.5004 **	0.4884 *	0.1108
TPL	0.2422	0.0769	− 0.3303	− 0.3755	− 0.0647	0.1762	− 0.201	0.19	0.2031	− 0.0178	0.1201	0.1044	− 0.1884
TFL	0.0409	0.4346 *	0.1808	0.1659	0.0321	0.0374	0.0437	0.3853	0.0211	0.0292	0.0151	− 0.0053	− 0.3197
CUPRACL	0.1128	− 0.0196	− 0.3945*	− 0.3214	0.2241	0.003	− 0.4759*	0.3099	0.0913	0.1891	0.2081	0.191	0.4197*
DPPHL	− 0.0886	− 0.2517	− 0.4566*	− 0.4223*	− 0.1226	− 0.102	− 0.4983**	− 0.2833	− 0.1794	− 0.1776	− 0.2084	− 0.2218	0.1198
FRAPL	− 0.0542	− 0.036	− 0.2237	− 0.0909	0.1607	− 0.1621	− 0.4118*	-0.0406	− 0.1335	0.1244	8.00E-04	0.0182	0.3532
TPF	0.1599	0.1001	-0.2758	-0.3237	0.4282*	-0.0584	-0.0839	0.0069	0.1845	0.4534*	0.4281*	0.3892*	-0.0448
TFF	0.1209	0.2506	0.0623	0.2106	− 0.0152	0.3204	− 0.3229	0.3212	0.4053*	− 0.005	0.2807	0.2399	0.1154
CUPRACF	0.0136	0.3697	− 0.3085	− 0.2769	0.1017	0.3518	− 0.1849	0.3563	0.286	0.1235	0.2558	0.1839	− 0.1433
DPPHF	− 0.1344	0.0607	− 0.2007	− 0.0612	0.529**	− 0.314	− 0.2367	0.0478	− 0.1774	0.5441**	0.2906	0.2856	0.2874
FRAPF	0.0134	0.1823	− 0.2259	− 0.1426	0.4166*	− 0.0634	− 0.3112	0.5214**	0.0572	0.3869	0.3208	0.2789	− 0.0069

Traits	Ac	TSS	TPL	TFL	CUPRACL	DPPHL	FRAPL	TPF	TFF	CUPRACF	DPPHF	FRAPF	
VL	0.2923**	0.2273*	0.2662*	0.0733	0.0966	− 0.041	− 0.0046	0.1944	0.1907	0.2088	0.0262	0.1001	
IL	− 0.2592*	− 0.0464	0.0781	0.4049**	− 0.0174	− 0.2441*	− 0.0403	0.0829	0.2275*	0.3039**	0.0431	0.1601	
LL	− 0.1084	− 0.0987	− 0.2781*	0.1465	− 0.3602**	− 0.4081**	− 0.2051	− 0.2478*	0.0592	− 0.2335*	− 0.1609	− 0.187	
LW	− 0.0512	− 0.0242	− 0.3016**	0.1282	− 0.2065	− 0.2782*	− 0.0584	− 0.2002	0.1429	− 0.1604	− 0.0694	− 0.0621	
NFFP	− 0.1807	0.2543*	− 0.0077	0.0224	0.1847	− 0.1171	0.1376	0.3506**	− 0.0088	0.0556	0.452**	0.3459**	
FL	0.0207	8.00E− 04	0.2079	0.0309	− 0.0047	− 0.0955	− 0.1439	− 0.0861	0.2932**	0.2755*	− 0.2452*	− 0.0596	
PL	− 0.2673*	− 0.1188	− 0.0988	0.0275	− 0.4282**	− 0.4439**	− 0.3645**	− 0.0915	− 0.2483*	− 0.1162	− 0.1505	− 0.2473*	
FD	− 0.018	− 0.1283	0.1203	0.3489**	0.2848*	− 0.231*	− 0.0192	0.0376	0.291**	0.365**	0.0453	0.4848**	
AFW	0.1362	0.1098	0.1274	0.0035	0.0704	− 0.1841	− 0.1494	0.1054	0.2558*	0.053	− 0.2632*	− 0.0329	
NFPY	− 0.2795*	0.2857*	− 0.0549	0.0245	0.1636	− 0.1793	0.0921	0.3688**	− 0.0698	− 0.0285	0.3847**	0.2787*	
FYP	− 0.0975	0.2819*	0.0374	0.0087	0.1564	− 0.1968	− 0.0312	0.2892*	0.1238	− 0.0053	0.0938	0.1591	
FY	− 0.0151	0.3746**	0.0695	− 0.0136	0.1784	− 0.2248*	− 0.0023	0.322**	0.1667	0.0434	0.1693	0.1915	
AsA	0.3639**	0.152	− 0.1533	− 0.2946**	0.4049**	0.1279	0.3572**	− 0.0201	0.1441	− 0.0437	0.3152**	0.0329	
Ac	1	− 0.0196	0.2595*	0.0181	0.1652	0.272*	0.2636*	0.1597	− 0.0723	0.043	0.132	− 0.0534	
TSS	− 0.1453	1	0.1051	− 0.0476	0.0699	0.042	0.0343	0.1377	0.1282	− 0.1125	0.2227	− 0.1224	
TPL	0.2595	0.0947	1	0.4971**	0.3811**	0.2693*	0.1384	0.5589**	0.2714*	0.5217**	0.1742	0.3151**	
TFL	− 0.0192	− 0.0663	0.5393**	1	0.305**	− 0.1534	0.1707	0.2928**	0.3348**	0.6118**	0.4368**	0.4771**	
CUPRACL	0.1895	0.0717	0.4073*	0.3293	1	0.3803**	0.6105**	0.3234**	0.4354**	0.3791**	0.4714**	0.4915**	
DPPHL	0.2801	0.0317	0.2707	− 0.1581	0.3807	1	0.5353**	0.2377*	− 0.0613	− 0.0087	0.1386	− 0.1513	
FRAPL	0.2589	0.0191	0.1372	0.1646	0.6199**	0.5372**	1	0.2808*	0.2167	0.102	0.5442**	0.2653*	
TPF	0.1274	0.113	0.5895**	0.2939	0.3331	0.2437	0.281	1	0.1445	0.3513**	0.3612**	0.2528*	
TFF	− 0.1933	0.0863	0.2595	0.348	0.4573*	− 0.0672	0.2135	0.1311	1	0.5663**	0.2063	0.583**	
CUPRACF	− 0.1736	− 0.2325	0.5645**	0.6548**	0.4275*	− 0.0321	0.0786	0.3434	0.551**	1	0.3109**	0.7116**	
DPPHF	0.0221	0.1812	0.1432	0.4641*	0.5013**	0.1356	0.5603**	0.3565	0.161	0.2439	1	0.3891**	
FRAPF	− 0.1666	− 0.1752	0.3268	0.4919*	0.5148**	− 0.1645	0.2629	0.2382	0.574**	0.7107**	0.3712	1	

Statistically significant correlations are denoted by an asterisk (*) where * P ≤ 0.05, ** P ≤ 0.01, and *** P ≤ 0.001.

### Principal component analysis (PCA)

**Figure 7:**
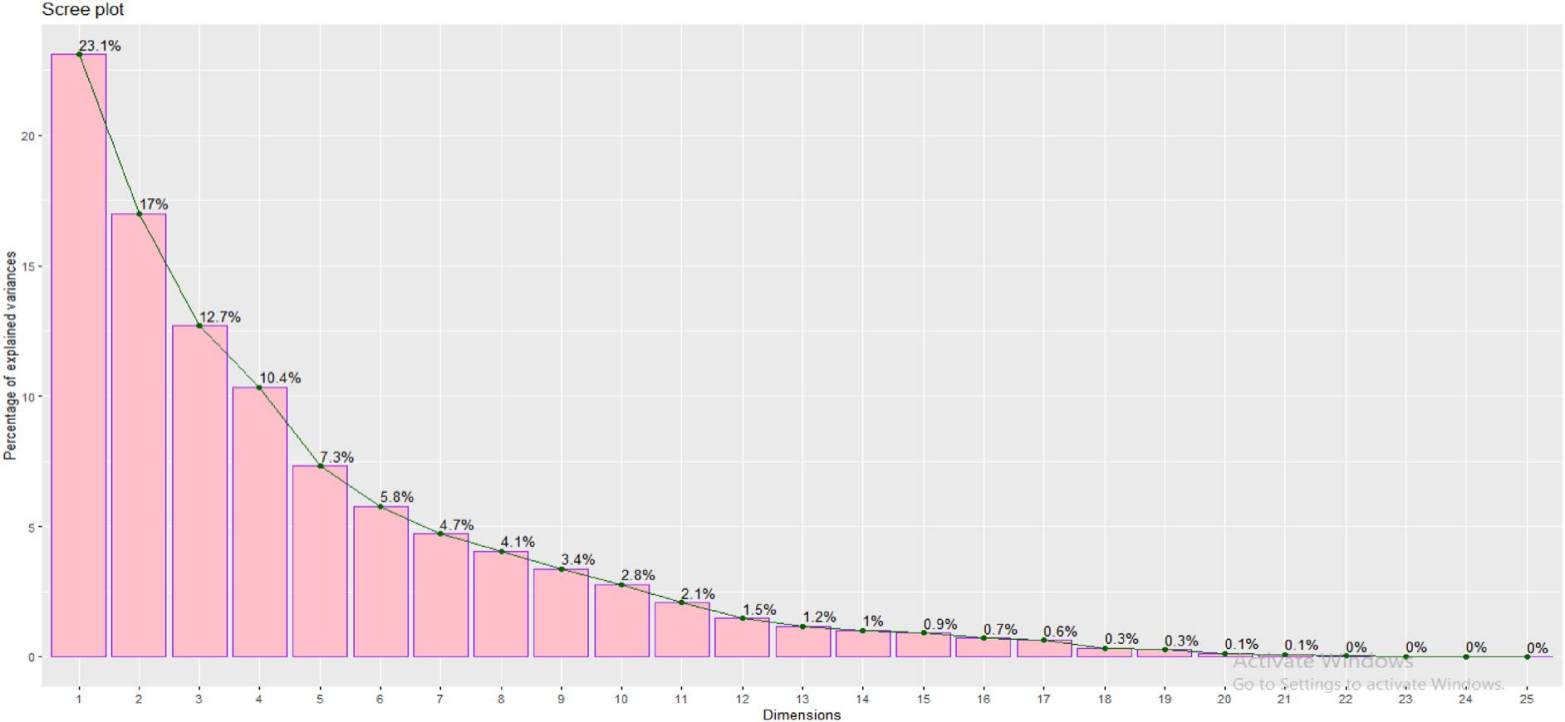
Scree graph for per cent variation explained by principal components based on 25 growth, yield and quality traits in 26 *coccinia* gynoecious accessions.

**Figure 8:**
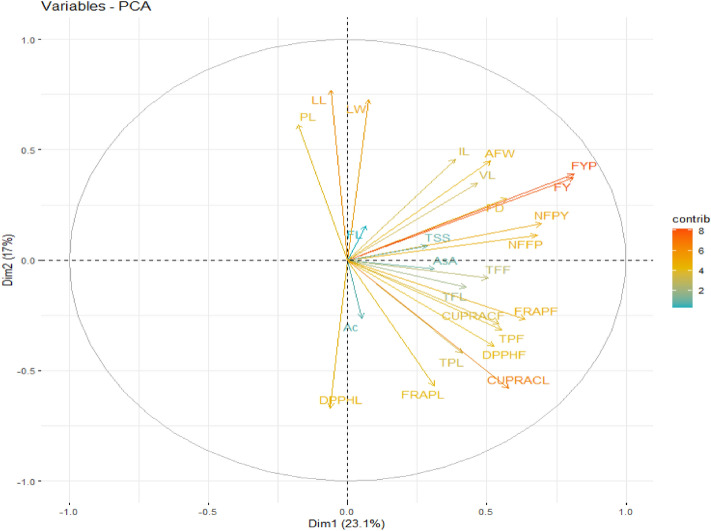
Characters per cent contribution towards the principal components to the total variation of for twenty five growth, yield and quality parameters in 26 *coccinia* gynoecious accessions.

**Figure 9:**
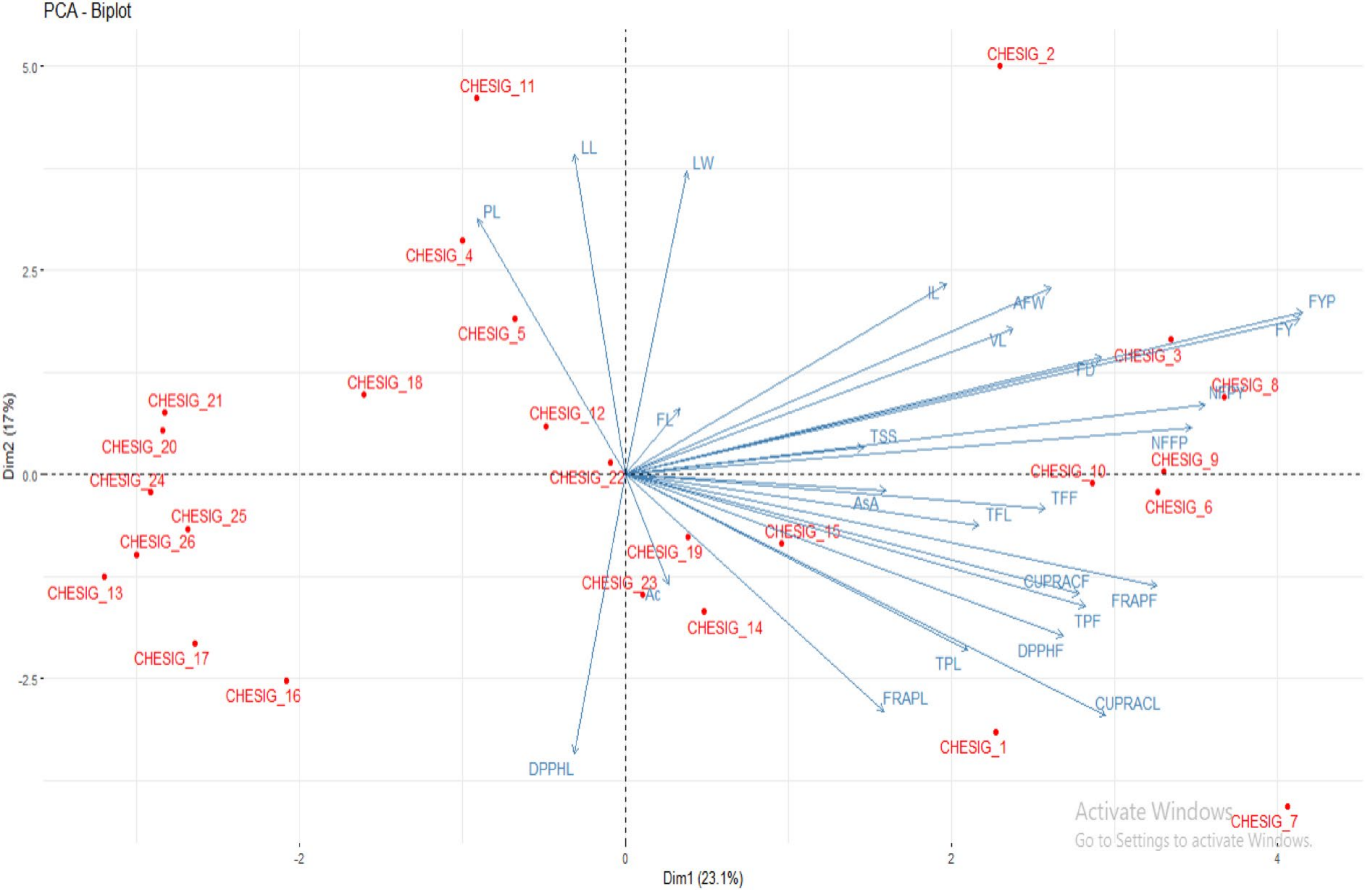
The PCA Biplot plot showing the 26 *coccinia* gynoecious accessions and their percent variation to the total variation for 25 traits.

The present study explains the PCA of 26 *coccinia* gynoecious accessions with 25 growth and yield as well quality parameters comprises the eight eigen values. The eigen values and their percent variation were presented (Tables 8, 9 and Figs. 7, 8, 9) Eigen value and variance connected with each PC gradually decreases but cumulative variability gradually increases (Table 8 and Fig. 7). The present study, eight PC-I to PC-VIII having eigen values greater than one and it comprises 85.02% of the total variation for 26 *coccinia* gynoecious accessions. The components and their eigen values accounts greater than one are considered as principal components (Major components), which are responsible for higher magnitude of variance.

**Table 8:** Eigen values, variation explained (%) and cumulative variance (%) of principal component analysis in Ivy gourd accessions.

S.N.	Principal component	Eigen value	Variation explained (%)	Cumulative variance (%)
1	PC-I	5.774607	23.09843	23.09843
2	PC-II	4.249762	16.99905	40.09748
3	PC-III	3.174752	12.69901	52.79649
4	PC-IV	2.587992	10.35197	63.14846
5	PC-V	1.828482	7.313929	70.46239
6	PC-VI	1.446917	5.787666	76.25005
7	PC-VII	1.178733	4.714934	80.96499
8	PC-VIII	1.012859	4.051437	85.01642

**Table 9:** Eigen vector, Eigen root and associated variation for principal component in Ivy gourd accessions on growth, yield and quality parameters.

Traits	PC1	PC2	PC3	PC4	PC5	PC6	PC7	PC8
VL	0.466	0.350	0.075	0.433	− 0.075	0.225	− 0.206	− 0.105
IL	0.386	0.458	0.411	− 0.188	0.093	0.176	0.198	0.185
LL	− 0.061	0.769	0.165	− 0.120	0.458	0.272	0.044	− 0.154
LW	0.074	0.727	0.109	− 0.112	0.579	0.196	0.092	− 0.151
NFFP	0.681	0.113	− 0.537	− 0.425	− 0.087	− 0.034	− 0.088	0.007
FL	0.065	0.156	0.463	0.615	− 0.186	− 0.222	0.319	0.289
PL	− 0.178	0.613	0.114	− 0.166	− 0.191	0.198	0.109	0.556
FD	0.572	0.280	0.344	− 0.071	0.062	− 0.125	− 0.470	− 0.079
AFW	0.511	0.448	0.097	0.671	− 0.018	− 0.010	− 0.020	− 0.043
NFPY	0.696	0.166	− 0.538	− 0.385	− 0.173	− 0.023	− 0.037	0.060
FYP	0.814	0.390	− 0.339	0.157	− 0.113	− 0.028	− 0.038	− 0.055
FY	0.810	0.373	− 0.352	0.191	− 0.137	− 0.045	− 0.017	− 0.007
AsA	0.313	− 0.038	− 0.414	0.468	0.412	− 0.278	0.057	0.433
Ac	0.051	− 0.263	− 0.104	0.582	0.238	0.380	− 0.380	0.037
TSS	0.286	0.065	− 0.432	0.140	− 0.216	0.049	0.614	− 0.362
TPL	0.411	− 0.422	0.411	0.186	− 0.354	0.416	0.020	− 0.091
TFL	0.425	− 0.123	0.629	− 0.344	0.093	0.346	0.106	0.014
CUPRACL	0.577	− 0.579	0.068	0.110	0.288	− 0.122	− 0.008	0.046
DPPHL	− 0.062	− 0.670	− 0.182	0.273	0.140	0.263	0.064	− 0.102
FRAPL	0.310	− 0.568	− 0.141	0.008	0.562	0.150	0.115	0.112
TPF	0.552	− 0.315	− 0.061	− 0.031	− 0.331	0.465	0.056	0.095
TFF	0.505	− 0.082	0.474	0.107	0.245	− 0.363	0.310	− 0.283
CUPRACF	0.545	− 0.287	0.639	− 0.091	− 0.199	− 0.159	0.016	0.121
DPPHF	0.526	− 0.389	− 0.202	− 0.372	0.295	0.136	0.208	0.228
FRAPF	0.639	− 0.267	0.346	− 0.335	0.041	− 0.359	− 0.213	− 0.055

The components with eigen values larger than one are considered as principal components or significant components since they account for a large proportion of the variance. Plant breeders typically pick such components for plant selection. Considering of such components will be more useful in the plant selection and further improvement of coccinia^11,13^. The eigen values of PC-I was comprised approximately 23.0984% of total variation followed by PC-II (16.999%), PC-III (12.699), PC-IV (10.351%), PC-V (7.313%), PC-VI (5.787%), PC-VII (4.714%) and PC-VIII (4.051%). It indicates that, presence of appreciable diversity among the genotypes for the characters under study. More or less similar results were recorded by^4,35,40–43^. The characters contribution towards the principal components for twenty five growth, yield and quality parameters were given in Table 9 and Figs. 8 and 9. The most of characters were positively contributed to the PC-I which are VL (0.466), IL (0.386), LW(0.074), NFFP (0.681), FL (0.065), FD (0.572), AFW (0.511), NFPY (0.696), FYP (0.814), FY (0.810), AsC (0.313), Ac (0.051), TSS (0.286), TPL (0.411), TFL (0.425), CUPRACL (0.577), FRAPL(0.310), TPF (0.552), TFF(0.505), CUPRACF (0.545), DPPHF (0.526) and FRAPF (0.639).Whereas character like LL (− 0.061), PL (− 0.178) and DPPHL (− 0.062) contributed negatively. This reveals that PC-I contributed the large amount (23.098%) of variability through its growth, yield and yield attributing and quality traits. Similarly in cucumber, the first five PCs showed an eigen value of 1 and above with 71.51% cumulative variance. Among these, the first two PCs accounted 43.92% cumulative variance and they were highly associated with the days to a male flower, days to a female flower, the number of nodes at the first female flower, the first fruit harvest, fruit length, fruit width, fruit weight, and the number of fruits per plant in cucumber^27^. Likewise in another study by^11^ in *Coccinia,* first three PCs accounted for 50% of the total variance and differences among the accessions were evidenced principally in relation to fruit traits such as fruit weight, fruit length and the number of seeds in each fruit. The main characters positively contributed to the PC-II were VL (0.350), IL (0.458), LL (0.769), LW (0.727), NFFP (0.113), FL (0.156), PL (0.613), FD (0.280), AFW (0.448), NFPY (0.166), FYP (0.390), FY (0.373) and TSS (0.065) were responsible for 18.24% of total variability. Whereas, all the quality parameters like, AsC (− 0.038), Ac (− 0.263), TPL (− 0.422), TFL (− 0.123), CUPRACL (− 0.579), DPPHL (− 0.670), FRAPL (− 0.568), TPF (− 0.315), TFF (− 0.082), CUPRACF (− 0.287), DPPHF (− 0.389) and FRAPF (− 0.267) contributed negatively to this component. IL (0.411), LL (0.165), LW (0.109), FL (0.463), Pl (0.114), FD (0.344), TPL (0.411), TFL (0.629), TFF (0.474), CUPRACF (0.639) and FRAPF (0.346) were major traits contributes positively for variation (12.699%) in the PC-III, while NFFP (− 0.537), NFPY(− 0.538), FYP (− 0.339), FY(− 0.352), AsC (− 0.414), Ac (− 0.104), TSS (− 0.432), DPPHL (− 0.182), FRAPL (− 0.141) and DPPHF (− 0.202) were negatively contributed. The major characters contributes positively for variation(10.351%) towards PC-IV were VL (0.433), FL (0.615), AFW (0.671), FYP (0.157), FY (0.191), AsC (0.468), Ac (0.582), TSS (0.140), TPL (0.186), CUPRACL (0.110), DPPHL (0.273) and TFF (0.107), while IL (− 0.188), LL (− 0.120), LW (− 0.112), NFFP (− 0.385), TFL (− 0.344), DPPHF (− 0.372) and FRAPF (− 0.335) were negatively contributed. Similar results were obtained by^13^ who studied morphological diversity of wild genetic resources of alfalfa and detected that the first PC explained 56.4% of the total variability in the measured traits and was associated with biomass production, which is congruent with our results. The PC-V is responsible for 7.313% of total variability through major characters positively contributed to this component were LL (0.458), LW (0.579), AsC (0.412), Ac (0.238), CUPRACL (0.288), DPPHL (0.140), FRAPL (0.562), TFF (0.245) and DPPHF (0.295) Whereas, FL (− 0.186), PL (− 0.191), NFPY (− 0.173), FYP (− 0.113), FY (− 0.137), TSS (− 0.216) TPL (− 0.354), TPF (− 0.331) and CUPRACF (− 0.199) were contributed negatively to this component. The major characters positively contributes for variation (5.787%) towards PC-VI were VL (0.225), IL (0.176), LL (0.272), LW(0.196), PL (0.198), Ac (0.380), TPL (0.416), TFL (0.346), DPPHL (0.263), FRAPL (0.150) and DPPHF (0.136), while the negatively contributed traits in this component were FL (− 0.188), FD (− 0.125), AsC (− 0.278), CUPRACL (− 0.122) TFF (− 0.363), CUPRACF (− 0.159) and FRAPF (− 0.359). In PC-VII, the major traits positively contributed (4.714%) to the total variability were IL (0.198), FL (0.319), PL (0.109), TSS (0.614), TFL (0.106), FRAPL (0.115), TFF (0.310), DPPHF (0.208) and negatively contributed traits were VL (− 0.206), FD (− 0.470), Ac (− 0.380) and FRAPF (− 0.213). The PC-VIII contributes only 4.05%.of total variability and the major characters responsible for this component were IL (0.185), FL (0.289), PL (0.556), AsC (0.433), FRAPL (0.112), CUPRACF (0.121) and DPPHF (0.228) whereas, negatively contributed traits were VL (− 0.105), LL (− 0.154), LW (− 0.151), TSS (− 0.362), DPPHL (− 0.102) and TFF (− 0.283). Character contributed positively towards PC-I to PC- VIII are important because their contribution is more than 85.02% of total variability.

The Similar, first two PCs accounted 81.27% of the total variation among for 38 brinjal lines for different traits^44^. The PCA revealed higher contribution for variation mainly comes from the characters like VL, NFFP, FL, FD, AFW, NFPY, AsC and TSS. These results were in line with the findings^13,35,38,40–42^.

### Cluster mean analysis

**Figure 10:**
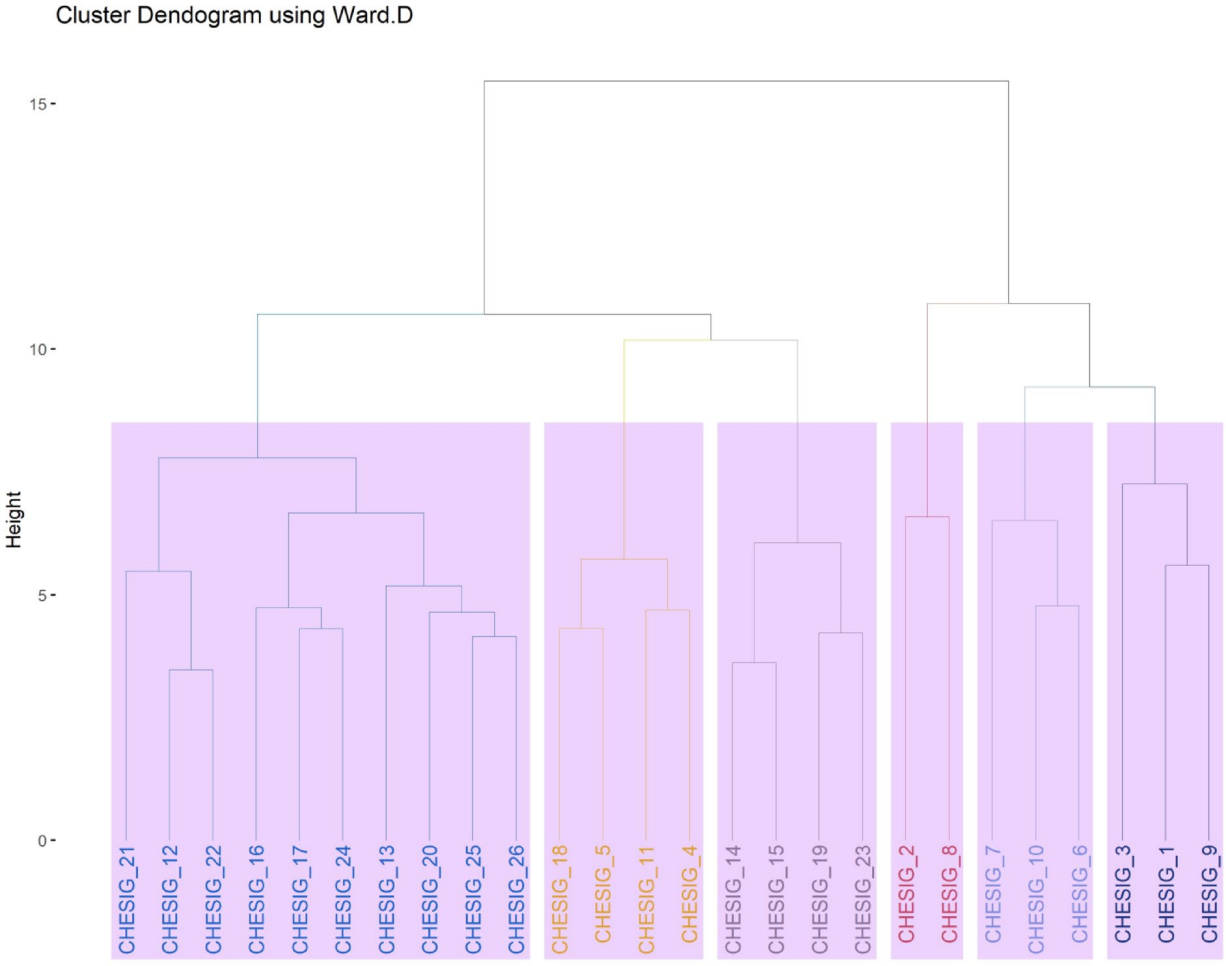
Dendrogram showing relationship among 26 coccinia gynoecious accessions.

**Figure 11:**
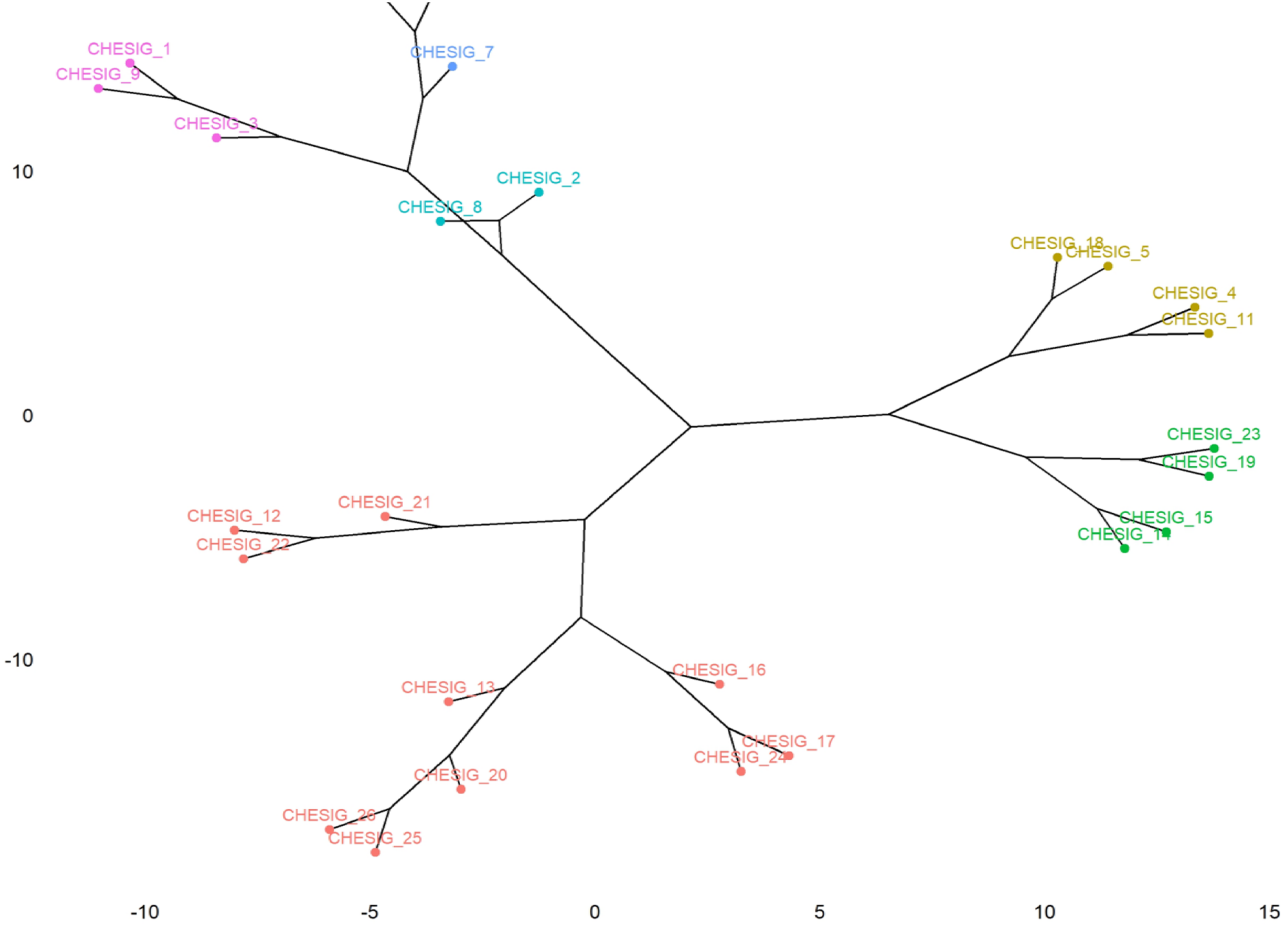
Cluster tree explains the composition of twenty six ivy gourd accessions in to six clusters.

Cluster mean analysis for twenty five traits in 26 ivy gourd accession for growth, yield and quality parameters were given in Table 2. The cluster composition of twenty six ivy gourd genotypes in to six clusters were also worked out (Table 3 and Figs. 10, 11). Clusters means analysis shows a wider variation among the growth and yield related parameters (Table 2) which could be due to different unique characteristics (Table 4). The neighbor-joining dendrogram is a tool for explaining objects, which has been widely used as an effective tool to discover the structural associations among tested accesions and provides a hierarchical classification of them. The higher mean values for VL (4.428) was observed in cluster VI followed by cluster I (349.6), while, lower cluster mean value was observed in cluster I I (294.150), The high cluster mean for IL was observed in cluster I (8.300) followed by cluster III (7.750), while the lowest was observed in cluster V (6.450). The LL has higher mean values in the cluster III (8.40) and clusters VI (8.30) whereas, low values in cluster (V). Highest cluster mean for LW was noticed in cluster VI (7.80) followed by cluster III (7.725) and lowest values in cluster V (6.525). The NFFP had the highest mean values in cluster II (1621.33) followed by cluster V (1453.75) while, the lowest in cluster IV (941.60). The high cluster mean for FL was recorded in cluster VI (6.350) and cluster I (6.100) with low mean in cluster II (4.533). The similar trend in cluster analysis bitter gourd genotypes for different traits showed that the line PDMGy 201 was distinct from the rest of the three genotypes in cluster I, which could be due to its unique characteristics, like gynoecious reproduction, and its superiority for earliness traits like the number of days until the first female flower appearance and the number of days until the first fruit is harvested^35^.

The maximum cluster mean for PL was recorded in cluster III (7.325) followed by cluster I (6.033), and minimum was recorded in cluster II (5.033).The FD has got high cluster mean values in cluster I (3.067) followed by cluster VI (2.800) while, low values in cluster IV (2.220).The AFW recorded the highest cluster mean values in cluster VI (24.750) followed by cluster I (18.330) and the lowest was recorded in cluster III (14.225). The high cluster mean values for NFPY was observed in cluster II (1465.667) and cluster V (1390.750) while, low value in cluster IV (844.700).The trait FYP got highest cluster mean value in cluster VI and II (31.418 and 22.899), whereas, low values in cluster IV (12.406). The higher mean values for FY (26.090) were observed in cluster VI followed by cluster II (18.265) and lower values in cluster I V (10.084). In line with these results, 103 landraces of cucumber grouped in six clusters and cluster III with landraces AC-14, AC-97, AC-471, AC-451, and RAI-209 were found more divergent for average fruit weight, fruit length, and fruit width, while cluster IV with landraces AC-201, TT-161, RAI- 217, RAI-215, and TRMR-103 were found more divergent for improving average vine length, internodes length, the number of primary branches plant^−1^, the number of fruits plant^−1^, and yield plant^−1^^27^. In general, the intra cluster distance shows lower values than the inter cluster distances. Therefore, the genotypes accounts within a cluster group were less divergent from one another. It is desirable to select genotypes from cluster showing high inter cluster distance as it indicates the wider genetic diversity present in the genotypes^11^. Thus, these diverse lines may be used in the future improvement programme in the coccinia forndevelopment and selection of improved variety.

### Quality parameters

The cluster I has highest cluster mean values in the traits like TPL (22.453), TFL (11.313), CUPRACL (32.840), TPF (14.338), TFF (7.080), CUPRACF (24.320), DPPHF (21.246) and FRAPF (29.197) and lower values in AsC (25.940). Polyphenolic and flavonoid compounds are the chemical structure contains multiple hydroxyl substituent’s on an aromatic ring. Due to their structure, polyphenol compounds are good electron and proton donors. They are capable to scavenge free radicals and reduce oxidative stress by transferring H-atom from their hydroxyl group(s) to free radicals^45^. The cluster II exhibited high mean values for AsC (47.287), TFL (9.250), CUPRACL (36.899), FRAPL (41.536), CUPRACF (20.330), DPPHF (23.683)) and FRAPF (29.644). The cluster III shown lowest cluster mean values for most of the quality parameters under study like, Ac (0.083), TPL (13.308), CUPRACL (23.462), DPPHL (14.293), FRAPL (27.314), CUPRACF (17.293) and FRAPF (25.011). The cluster IV exhibited higher mean value for Ac (0.125), DPPHL (19.422) and lower values for TSS (1.560) and TPF (10.593). The cluster V has highest cluster mean values in TSS (2.125), TPL (19.633), DPPHL (20.883), FRAPL (30.458), TPF (13.810) and lower values for TFF (4.5528).The cluster VI exhibited higher cluster mean values for AsC (47.325), Ac (0.137), TSS (2.60) and TFF (6.615) whereas, lower values for TFL (6.540) and DPPHF (18.047). In previous studies of *Coccinia* (41) reported polyphenolic content of 104:88 ± 0:8 mg GAE equivalent per gram of leaf extract and flavonoids content of 35:35 ± 1:82 mg QE equivalent per gram of leaf extract. The accessions with higher mean values are having great scope while selecting the genotypes for further improvement in ivy gourd. The cluster VI and cluster I shown the valuable characters for attaining high fruit yield as well as quality in ivy gourd. The cluster VI has higher mean values for most of the traits viz.,VL, LL, LW, FL, FD, AFW, FYP, FY, AsC, Ac, TSS and TFF. Meanwhile, cluster I was found superior for VL, IL, FL, PL, FD, AFW, TPL, TFL, CUPRACL, TPF, TFF, CUPRACF, DPPHF and FRAPF. Thus, genotypes from the distinct cluster like cluster VI and I should be used for selection of parents and varietal improvement for further breeding programme in ivy gourd. The similar trend was presented by^4,5,11,13,30,35,38,39,41,43^. In fact, these researchers have compared their data with previously published database or literature which might have different genotype. Among the antioxidants, particularly ascorbic acid is thermolabile in nature and many of the vegetable at mature stages are generally consumed after cooking, the human body is unable to harness the benefits of ascorbic acid. On the other hand, the human body can fully access the ascorbic acid present in *coccinia* fruits by consuming it as fresh or table purpose^14^.

## Conclusion

Range of variation observed for all the traits which indicate the presence of sufficient amount of variation among the genotypes for all the characters. High heritability coupled with high Genetic advance as per cent meanwas observed for VL, IL, NFFP, FL, PL, FD, AFW, NFPY,FYP, FY, AsC, Ac, TSS, TPL, TFL, CUPRACL, DPPHL, FRAPL, TPF, TFF, CUPRACF, DPPHF, CUPRACF and DPPHF indicated additive gene action, making selection for this trait will be more effective. Most of the characters showed high positive correlation both in phenotypic as well as genotypic level, which indicating that there was simultaneous selection for these characters might bring an improvement in fruit yield of plant. Eight principal components PC-I to PC-VIII having eigen values greater than one and it comprises 85.02% of the total variation. The PCA revealed higher contribution for variation mainly comes from the characters like VL, NFFP, FL, FD, AFW, NFPY, AsC and TSS. The cluster VI and cluster I shown the valuable characters for attaining high fruit yield as well as quality in ivy gourd. The cluster VI has higher mean values for most of the traits viz.,VL, LL, LW, FL, FD, AFW, FYP, FY, AsC, Ac, TSS and TFF. Meanwhile, cluster I was found superior for VL, IL, FL, PL, FD, AFW, TPL, TFL, CUPRACL, TPF, TFF, CUPRACF, DPPHF and FRAPF. These results are based on 25 important traits to assess the 26 gynoecious accessions of *coccinia* for selection of trait specific lines in the present experiment which will be helpful in enhancing the productivity and availability to the consumer’s along with the exploitation of natural genetic variation existing in the available germplasm. Thus, genotypes from the distinct cluster should be used for selection of parents and varietal improvement for further breeding programme in ivy gourd.

## Data Availability

The datasets used and/or analyzed during the current study available from the corresponding author on reasonable request.
